# Proteolysis and therapeutic potential of bioactive peptides derived from Cheddar cheese

**DOI:** 10.1002/fsn3.3501

**Published:** 2023-06-13

**Authors:** Bakhtawar Shafique, Mian Anjum Murtaza, Iram Hafiz, Kashif Ameer, Shahnai Basharat, Isam A. Mohamed Ahmed

**Affiliations:** ^1^ Institute of Food Science and Nutrition University of Sargodha Sargodha Pakistan; ^2^ Institute of Chemistry University of Sargodha Sargodha Pakistan; ^3^ The University Institute of Diet and Nutritional Sciences The University of Lahore Lahore Pakistan; ^4^ Department of Food Science and Nutrition, College of Food and Agricultural Sciences King Saud University Riyadh Saudi Arabia; ^5^ Department of Food Science and Technology, Faculty of Agriculture University of Khartoum Shambat Sudan

**Keywords:** anti‐hypertensive, anti‐oxidant, bioactive peptides, Cheddar cheese, proteomics, therapeutic aspect

## Abstract

Cheddar cheese‐derived bioactive peptides are considered a potential component of functional foods. A positive impact of bioactive peptides on diet‐related chronic, non‐communicable diseases, like obesity, cardiovascular diseases, and diabetes, has been observed. Bioactive peptides possess multifunctional therapeutic potentials, including antimicrobial, immunomodulatory, antioxidant, enzyme inhibitory effects, anti‐thrombotic, and phyto‐pathological activities against various toxic compounds. Peptides can regulate human immune, gastrointestinal, hormonal, and neurological responses, which play an integral role in the deterrence and treatment of certain diseases like cancer, osteoporosis, hypertension, and other health disorders, as described in the present review. This review summarizes the categories of the Cheddar cheese‐derived bioactive peptides, their general characteristics, physiological functions, and possible applications in healthcare.

## Graphical abstract

Cheese‐derived bioactive peptides are considered a potential component of functional foods. Bioactive peptides possess multifunctional therapeutic potentials. Peptides can regulate human immune, gastrointestinal, hormonal, and neurological responses.

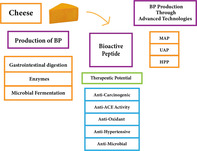

## 1 INTRODUCTION

For cheese production, milk is coagulated, and casein is concentrated at the right temperature and pH (Murtaza et al., 2014). To produce natural cheese, apposite starter cultures are used for acid production while rennet coagulates the casein micelles in the milk. Generally, strains from mesophilic bacteria are selected culture for cheese production. Cheese variety, processing technology, and ripening time, temperature are considered during the selection of starter cultures (Lollo et al., 2013; Murtaza, Anees‐Ur‐Rehman, et al., 2022).

Cheese is a healthy and nutritious food presented in a variety of flavors, textures, and forms. In the global market, it is available in more than 2000 varieties of different tastes, flavors, and forms (Almena‐Aliste & Mietton, 2014; Irfan et al., 2023). Ripened type cheese varieties like Cheddar cheese possess zero or reduced quantities of lactose. Being a calcium‐enriched food, this could be a significant source of calcium for the lactose‐intolerant community (Juan et al., 2016).

Moisture contents, manufacturing technology, ripening time, temperature, cheese composition are the prime factors upon which cheese varieties are classified (Markey et al., 2017). In the cheese group, Cheddar takes place in hard ripened types. It consists of a complex mixture of nutrients (e.g., protein, fat, carbohydrates, peptides, fatty acids, and amino acids), minerals (e.g. zinc, calcium, phosphorous), and vitamins (e.g., vitamin A, riboflavin, and vitamin B12) (Murtaza et al., 2014). A series of microbiological, metabolic, and biochemical reactions like glycolysis, lipolysis, and proteolysis occurs during ripening of Cheddar cheese. As a result of these reactions, the flavor and texture of Cheddar cheese develop (Jia et al., 2021; Murtaza, 2016).

Probiotics are living microbes and are known to positively affect health when taken in prescribed amounts, besides their inherent nutrition. The therapeutic potential of probiotics in certain diseases like gastrointestinal disorders, cancer, hyperlipidemia, allergies, liver diseases, lactose intolerance, and cholesterol assimilation is understood (Azat et al., 2016; Raza et al., 2021).

The addition of suitable probiotic strains in fermented dairy products promote peptide formation. Fermented dairy products, for example, yogurt, cheese, cultured buttermilk, sour cream, and kefir, are considered the most suitable foods for effective delivery and viability of probiotic strains (Meira et al., 2015). Cheese is characterized by higher levels of pH, total solids, and fat than other fermented dairy products (Chaves & Gigante, 2016). Two genera, namely *Lactobacillus* and *Bifidobacterium*, are considered for their excellent probiotic bacterial potential (Fernández‐Tomé et al., 2019).

The lactobacilli are non‐flagellated Gram‐positive bacteria. Some species of lactobacilli are aerotolerant, while the rest are non‐aerobic. Enzyme flavoprotein oxidase assists the aerotolerant species of lactobacilli during the utilization of oxygen. The recorded growth rate of lactobacilli observed ranges from 5.5 to 5.8 pH (Pitigraisorn et al., 2017). Lactobacilli species are commonly used as starter culture or probiotic adjuncts during the manufacturing of different food products. Lactobacilli facilitate the conversion of lactose into lactic acid. Lactic acid is easily digested by lactose‐intolerant people (López‐Expósito et al., 2017). Gastrointestinal tract pH is reduced due to the activity of lactobacilli. Reduced pH of the gastrointestinal tract inhibits the growth and endurance of putrefactive microbes (Pastrana et al., 2017). *Lactobacillus* spp. produces such compounds that are named bacteriocins along with metabolized hydrogen peroxide, carbon dioxide, and diacetyl which contain antimicrobial properties opposite to Gram‐positive as well as Gram‐negative bacteria (Chaves & Gigante, 2016).

*Bifidobacterium* is a Y‐shaped rod, Gram‐positive bacteria. Thirty species of genus *Bifidobacterium* are categorized as probiotic strains. Out of thirty, ten species (e.g., *B. bifidum, B. adolescentis, B. breve, B. lactis, B. infants*, and *B. longum*) are isolated from the gastrointestinal tract of human beings, while the rest of the species are derived from the GIT of animals. Probiotic strains obtained by humans are capable of colonizing and remain viable easily in the human gastrointestinal tract; hence they are preferred to use in the dairy industry for the processing of probiotic dairy products (Aizawa et al., 2016).

Probiotic strain growth patterns and optimum growth conditions are of great concern during selecting probiotic strain for product development. The strain should be viable in the product during its consumption (Bustamante et al., 2017). Naturally, bifidobacteria grow in the absence of oxygen. The suitable temperature and pH for the growth of bifidobacteria are recorded from 37°C to 41°C and 6.5 to 7.0, respectively (Aizawa et al., 2016).

In human beings, the therapeutic potential of bifidobacteria is expressed by host immune system modulation, boosting the resistance against infectious diseases, controlling inflammatory bowel disease, and cancer prevention (Sultan et al., 2018). Some of the bifidobacteria species are capable of boosting the immunoglobulin production, assimilate the un‐metabolized food substrate, ultimately increasing the nutritional value of food, improving anticarcinogenic capacity and folic acid synthesis (Martinez‐Villaluenga et al., 2017).

Hydrolysis of dairy proteins produces extensive bioactive peptides that exhibit significant functions for the maintenance of human health. Recently, a new approach has been developed to produce bioactive peptides through advanced technologies like high pressure processing (HPP), microwave‐assisted processing (MAP), and ultrasound‐assisted processing (UAP) (Johnson et al., 2023; Mohanty et al., 2016). These innovative and environmentally friendly technologies are used in combination with enzymatic hydrolysis and fermentation process. The treatment of advanced technologies plays a key role to bring modification in the profile of peptides to increase the therapeutic potential (Murtaza, Irfan, et al., 2022).

Bioactive peptides contain specific protein fragments and provide many positive aspects to regulate the body's functions (Almena‐Aliste & Mietton, 2014). They attribute therapeutic aids to particular aims, lowers the toxicity level, structural diversity, and less aggregation in body tissues because of high properties of bio‐functionality and bio‐specificity (Mohanty et al., 2016). Milk proteins are associated with the enrichment of bioactive peptides. Bioactive peptides consist of 3–20 amino acids per molecule on average. When their precursor protein sequence breaks down from the original substance of protein, they become active (Ong & Shah, 2009). This review highlights the potential bioactivity of peptides derived from Cheddar cheese and discusses their important role in the control and prevention of several diseases.

## 2 SELECTION CRITERIA FOR PROBIOTICS

**FIGURE 1:**
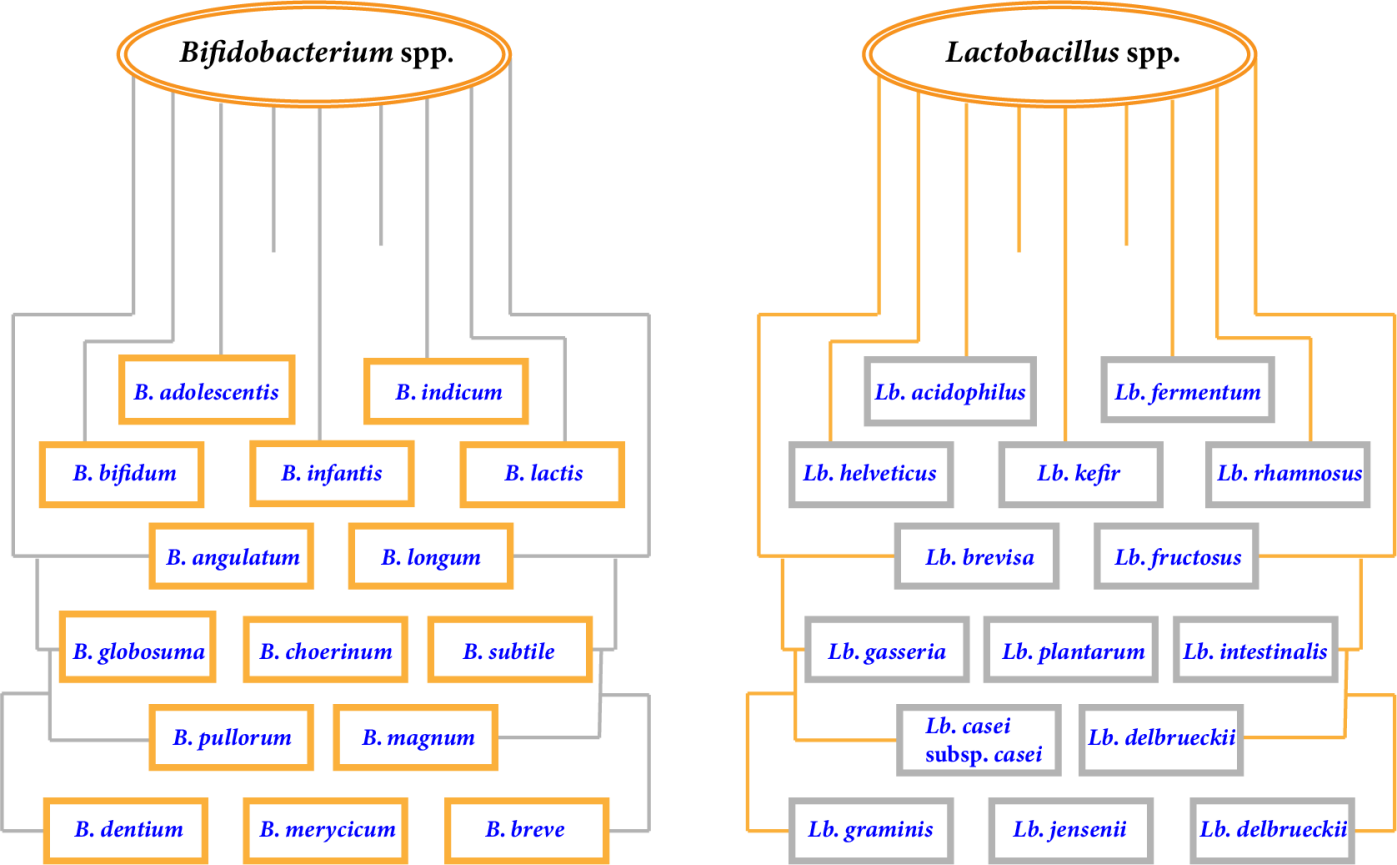
*Bifidobacterium* and *Lactobacillus* strains declared as GRAS status.

For food items, the probiotic strains are selected, keeping in view its safety, technological, functional, and physiological characteristics. *Lactobacillus* and *Bifidobacterium* strains are considered safe (GRAS) and help to reduce the cause of diseases. Qualified Presumption of Safety (QPS) status has been granted to these strains by the European Food Safety Authority (Pradhan et al., 2020). The GRAS probiotic strains are shown in Figure 1. For food, the human‐derived probiotic strains are preferred because only such strains can properly colonize and remain viable in the human gastrointestinal tract without imparting any toxic effect on human body (Keservani et al., 2017; Sheikh et al., 2023).

Certain fruits, vegetables, meat, fish, and legumes could also be a source of probiotic strains. Apart from the gastrointestinal tract of humans, some of the probiotic strains could be traced from other body parts like the oral cavity, urogenital tract, etc. (Ruiz‐Moyano et al., 2012). The adverse health‐related effects of the probiotic strains could not be ruled out. Their toxicity might be introduced by diffusion into blood cells or oral means (Bunesova et al., 2015).

A lot of in vivo and in vitro experiments for probiotic toxicity and safety are available in the literature (Farnworth & Champagne, 2016; Shafique et al., 2022). To ensure probiotic food safety, regulatory bodies have formulated their standards for probiotic selection. The daily recommended dose for probiotic strains in food is very high (10^9^ CFU/day). Any negligence in the selection of probiotic strains may cause severe health concerns. So, one should be very careful when selecting probiotic strains, especially for new strains, whose safe history is not available (de Melo Pereira et al., 2018).

WHO and FAO have proposed main criteria for the selection and screening of probiotics. A probiotic strain should colonize and survive in the gastrointestinal tract of host. Probiotics must contain cell surface properties, that is, cell surface hydrophobicity, auto‐aggregation, and ought to tolerate bile salt and low stomach pH (Lo Curto et al., 2011; Riaz et al., 2019). Moreover, probiotics should have functional properties, that is, antioxidant, antagonistic activities and cholesterol assimilation, to impart potential health benefits to the host (Ren et al., 2014). The probiotic's safety evaluation such as blood hemolytic activity, cytotoxicity, resistance to antibiotics, intestinal inflammatory cytokines, histopathological test, and antimutagenic activity should be performed besides functional attributes, before its interaction with the human or an animal. Recently, research on the antimicrobial activities of probiotics has been made as a primary concern in the control of food spoilage and pathogenic microorganisms. The efficacy of bifidobacteria to limit the occurrence of pathogenic bacteria has been well researched and studied (Gao & Li, 2018).

One of the safety requirements is the lack of hemolytic activity which is considered while making a selection of probiotic strain (FAO/WHO, 2002). Resistance to antibiotics is the major consideration regarding probiotic safety due to the ability of transferring antibiotic resistance genes to pathogenic bacteria in the habitat of intestine, which could show a risk for the infected patient's treatment. Antibiotic resistance is demonstrated as a negative feature for probiotics (Lee et al., 2014). The assessed strains revealed good resistance to gastric juice with concerned potential characteristics of probiotic (Wu, He, & Zhang, 2014).

General selection criteria for human food‐grade probiotic strains according to all available standards like QPS, European Union Product Safety Forum of Europe, World Health Organization, International Life Science Institute, Health Canada, US Food and Drug Administration Authority and Ministry of Health and Welfare Japan are summarized in Table 1.

**TABLE 1:** General considerations during probiotic strain selection for food.

Sr. No	Attributes	Considerations
01	General	Source of strain
		Antibiotic confrontation
		Metabolic pathway
		Pathogenic characteristics of strain
		Possible toxicity caused by strains
		Infection history of strains
02	Safety	Lack of hemolytic activity
		Resistance to antibiotics
		Presence of virulence genes
		Genes encoding antibiotic resistance
		Adherence to epithelial cells
		Good resistance to simulated gastric juice
03	Technological	Strain behavior at mass production
		Sensory attributes of strain
		Viability and stability during storage
		Viability and stability during processing
		Genetic structure and stability in food
		Phage resistance of probiotic adjunct
04	Physiological	Anti‐cancer characteristics
		Capacity to inhibit gastrointestinal pathogens
		Cholesterol assimilation of strain
		Lactose metabolism characteristics
		Anti‐mutagenic behavior
		Immunomodulation capacity of strain
05	Functional	Capacity to withstand in bile environment
		Capacity to withstand gastric pH
		Mucosal surface adherence
		Health impact of strain

## 3 BIOACTIVE PEPTIDES

In Cheddar cheese, protein (casein) varies from 4 to 40%, depending upon the variety. Most of its functional, nutritional, physicochemical, and sensory attributes are associated with protein components. During the ripening of Cheddar cheese, a series of enzymes originating from starter culture, probiotic adjuncts, rennet, non‐starter lactic acid bacteria participate in decomposing casein protein into peptides, amino acids, and other metabolites, making it almost 100% digestible (Garbowska et al., 2016). Quality, quantity, activity, and type of Cheddar cheese‐derived bioactive peptides depend upon pH, type of released enzymes, cheese composition, ripening time, temperature, and other ripening conditions (Ameer et al., 2020; Rodriguez‐Serrano et al., 2014). Probiotic adjuncts increase the proteolytic activity of Cheddar cheese (Murtaza et al., 2017) and improve the bioactivities of peptides (Ong & Shah, 2009).

Bioactive peptides are proteinous fragments having a hormonal or therapeutic effect on body functions (Taghizadeh et al., 2021). They are characterized by higher bio functionality and specificity, reduced toxicity, structural diversity, and reduced decomposition in the human body (Mohanty et al., 2016). Bioactive peptides are abundantly present in milk protein. One molecule of bioactive peptide contains 3–20 amino acids in composition (Mohanty et al., 2016). Bioactive peptides remain inactive in the precursor protein molecule and start their bioactivity soon after release from precursor protein (De Noni et al., 2009). The structural sequences of Cheddar cheese‐derived bioactive peptides amino acids are represented by one letter code in Table 2. The following methods release the encrypted bioactive peptides:

**TABLE 2:** Structural sequence of Cheddar cheese‐derived bioactive peptides amino acids represented by one letter code.

Bioactive peptide protein	Amino acid sequence	Structural sequence of bioactive peptide	Molecular weight (g.Mol^−1^)	Health promoting role	Reference
Lactoferricin	FKCRRWQWRMKKLGAPSITCVRRAF	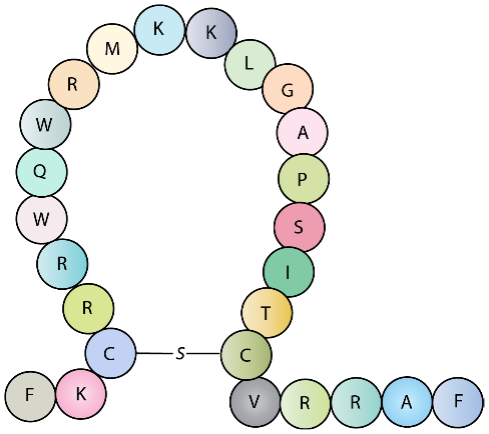	3125.8	Possess antimicrobial, antioxidant activities and inhibit the tumor formation that leads to anticarcinogenic effect	Bielecka et al. (2022)
α_s1_‐CN (f 1–9)	RPKHPIKHQ	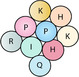	1140.7	Acts as immunopeptide and exhibits antimicrobial properties as well	Atanasova et al. (2021)
α_s1_‐CN (f 1–7)	RPKHPIK	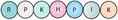	877.0	Anti‐thrombotic and mineral binding activity	Mehla (2020)
α_s1_‐CN (f 1–6)	RPKHPI	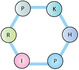	745.4	Opiate‐like peptide provides anti‐oxidative, anti‐hypertensive and antimicrobial properties	Chai et al. (2020)
β‐CN (f 47–52)	DKIHPF	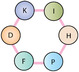	755.4	Imparts opioid, immunomodulatory and anti‐microbial properties	Kleekayai et al. (2021)
α_s1_‐CN (f 24–32)	FVAPFPEVF	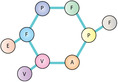	1053.3	Provides beneficial cardio‐protective properties by maintaining blood pressure	Mirzapour‐Kouhdasht and Garcia‐Vaquero (2022)
α_s1_‐CN (f 102–110)	KKYKVPQLE	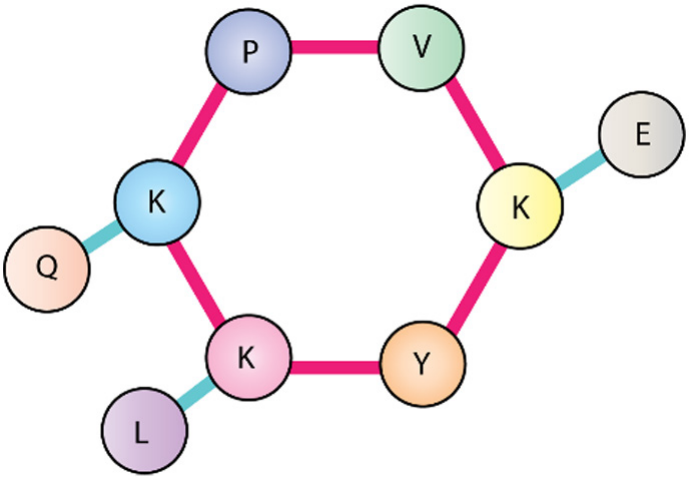	1132.4	Improves anti‐ACE activity for the maintenance of anti‐hypertensive potential	Hao et al. (2021)
β‐CN (f 193–209)	YQEPVLGPVRGPFPIIV	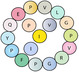	1881.1	Increases the inhibitory activity angiotensin converting enzyme	Giacometti Cavalheiro et al. (2020)
κ‐CN (f 96–102)	ARHPHPH	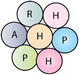	851.7	Improvement in digestibility and prevention of gastrointestinal tract infection	Zhang et al. (2022)

### 3.1 Gastrointestinal digestion (in vivo)

Pepsin, trypsin, and chymotrypsin (digestive enzymes) facilitate in vivo releasing the encrypted bioactive peptide during gastrointestinal digestion. In the stomach, hydrochloric acid (HCL) denatures the dietary protein and converts pepsinogen into pepsin. Pepsin is an active form of pepsinogen (Ramachandraiah et al., 2022). So, pepsin metabolizes denatured dietary protein into its amino acid fragments. Ultimately, dietary protein is hydrolyzed in the small intestine due to the role of digestive enzymes during gastrointestinal digestion (Das et al., 2020; Korhonen, 2009).

Antibacterial, antihypertensive, opioid, and immunomodulatory peptides released during gastrointestinal digestion of casein and whey proteins. Some of the proteolytic enzymes (thermolysin and pepsin) hydrolyze protein to produce ACE inhibitory, antioxidant, and antibacterial peptides during gastrointestinal digestion (Liu et al., 2018; Raza et al., 2022).

### 3.2 Microbial fermentation (in vitro)

Bioactive peptides are released by lactic acid bacteria (LAB) in the form of endo‐peptidases, amino‐peptidases, di‐peptidases, and tri‐peptidases during fermentation. During microbial proteolysis, a series of bioactive peptides have been reported to produce antioxidant, anti‐thrombotic, antihypertensive, opioid, antibacterial, and immunomodulatory peptides (Smacchi & Gobbetti, 2000; Zhao et al., 2021). The use of probiotic adjuncts facilitates the production of bioactive peptides. Probiotic adjuncts are recommended to use with the combination of mesophilic starter culture in Cheddar cheese (Jiang et al., 2022).

### 3.3 Enzymatic activity/processing conditions

Hydrolysis of milk protein through enzymes is a known way to activate the encrypted protein. Digestive enzymes, in combination with some bacterial and fungal proteinases, are capable of producing bioactive peptides from multiple proteins (Phelan & Kerins, 2011). Different processing techniques along with time–temperature varieties may also facilitate the bioactive peptide formation (Martinez‐Villaluenga et al., 2017).

### 3.4 Peptide absorption and stability

Oral intake of bioactive peptides is not adequate. Specific intracellular and extracellular barriers (like proteinases and peptidases) are present in the body, which reduces their effective absorption into the bloodstream (Jiang et al., 2021). The stomach environment is highly acidic (2–3 pH), while the pH of the duodenum ranges from 6 to 8. The abrupt change in pH may precipitate the peptides or protein. Endo, oxy, and carboxypeptidases of the pancreas may split the protein structure (Madureira et al., 2007).

The peptides and proteins are transported across epithelia by carrier‐induced pathways and by paracellular diffusion (Anusha & Bindhu, 2016), particularly cell uptake mechanisms transport peptides from apical to basolateral. The diffusion of peptides between adjacent cells is done in the paracellular route. Peptide degradation can be avoided by adopting carrier‐induced pathways and by paracellular diffusion (Wang et al., 2019).

The bioactive peptides are absorbed in the gastrointestinal tract of a human. Bicarbonate ions and glycoproteins are present on mucal lines of the gastrointestinal tract. 20–40 villi/mm^2^ are present at mucal lines having capillary blood vessels at its upper points. Nervous, lymphatic, and blood vessels are present at the submucosal space (space between mucus and apical surfaces). Peptides, after crossing epithelial cells, enter in venous system (Akkasheh et al., 2016; Feng et al., 2022).

Particular chemicals (oxidation, deamination, hydrolysis, etc.) and physical interactions may affect the stability of bioactive peptides. Oxidation of peptides is one of the major concerns affecting the strength of peptides. Encapsulation is considered the best technique to protect peptides from oxidation (Martins et al., 2007).

## 4 ROLE OF CHEDDAR CHEESE‐DERIVED BIOACTIVE PEPTIDES IN HUMAN HEALTH

**FIGURE 2:**
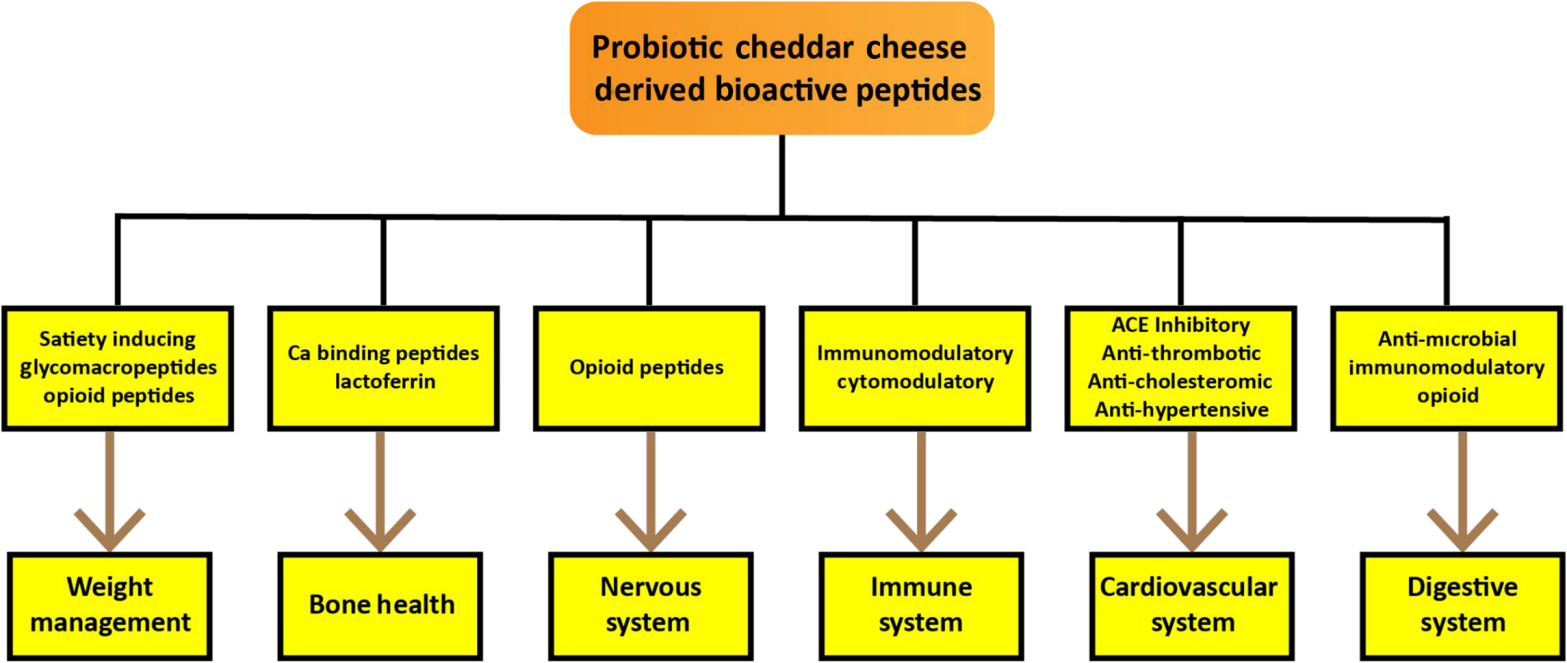
Probiotic Cheddar cheese‐derived bioactive peptides and their functionalities.

Bioactivities of probiotic cheese peptides depend upon the composition and arrangement of its encrypted amino acids. Opioid, anti‐thrombotic and antihypertensive (Seppo et al., 2003), immunomodulating, anti‐oxidative (Fernández‐Tomé et al., 2019), antimicrobial, anti‐cancer, mineral‐reserving and growth‐inducing functions are major bioactivities of peptides induced from probiotic Cheddar cheese (Verruck et al., 2019). The bioactivity of Cheddar cheese‐derived peptides occurs in the intestinal tract or inside the body after absorption (Erkaya & Şengul, 2015). The major functionalities of probiotic Cheddar cheese‐derived bioactive peptides are shown in Figure 2. The major bioactivities of peptides induced from Cheddar cheese are described below.

### 4.1 Antimicrobial peptides

Microbial growth‐inhibiting peptides in Cheddar cheese was firstly observed in 1930. Antimicrobial peptides are capable of eliminating or inhibiting the growth of microbes. β‐lactoglobulin, α_s1_‐casein, α‐lactalbumin, and к casein and other types of milk proteins are fundamental sources of antimicrobial peptides (Mohanty et al., 2016).

The majority of studies to access antimicrobial activities of probiotic Cheddar cheeses have been conducted on their water‐soluble extracts (WSEs). Along with peptides, organic acids are also present in water‐soluble extracts of cheese, so the exact quantification of antimicrobial peptides is not possible by this method. Purification of peptides is necessary to quantify the antimicrobial peptides present in probiotic Cheddar cheese. Antimicrobial activities of cheese varieties increase with ripening time. Maximum antimicrobial activities were observed after 6 months of ripening (Lignitto et al., 2012).

Théolier et al. (2014) accessed five commercial cheese varieties (old and medium Cheddar, Swiss, Mozzarella, and Gouda) with their water‐soluble extracts. Growth inhibition of *Listeria ivanovii* HPB 28, *Listeria monocytogenes* scot A3, *E. coli* MC4100 in water‐soluble extracts of Mozzarella and Gouda cheese was observed to 4.66 log cycles (Théolier et al., 2014).

Pritchard et al. (2010) studied the antimicrobial activity of commercially available three Australian Cheddar cheese varieties against *E. coli*, *B. cereus*, and *S. aureus*. Significant inhibition of all three microbes was observed as compared to control tryptone water. Shobharani and Agrawal (2011) accessed the antimicrobial effect of Cheddar cheese having *Leuconostoc paramesenteroides* against various food‐borne pathogens (like *Bacillus cereus, Listeria seeligeri, Listeria monocytogenes, Salmonella typhimurium*, and some others). The antimicrobial effect of isracidin and lactoferricin peptides were broadly studied against *Listeria monocytogenes* and *S. aureus* (Zhang et al., 2016). Further studies revealed that isradicin and lactoferricin peptides could inhibit and suppress the in vitro growth of *Lactobacillus* spp. and Gram‐positive bacteria (López‐Expósito et al., 2017).

### 4.2 Anti‐tumor/anti‐cancer peptides

Cancer is the second primary disease leading to deaths in developing countries. Chemotherapy, surgery, and radiotherapy are treatments used for cancer. Inadequate response and specific health problems are reported by these therapies (Sah et al., 2015). Milk‐derived bioactive peptides possess anti‐cancer characteristics. The peptides capable of inhibiting or suppressing cell proliferation both in vivo or in vitro are called anti‐tumor peptides (Balaji Raja & Arunachalam, 2011). Anti‐tumor peptides play a pivotal role in inhibiting the growth of tumors. Both milk proteins, casein and whey, contain anti‐tumor peptides. In human and animal cancer cell lines, anti‐tumor peptides cause apoptosis and necrosis. Multiple studies show that opioid peptides participate in an anti‐tumor activity mechanism (Songisepp et al., 2012).

Various studies are available on anti‐tumor/anti‐cancer properties of probiotic Cheddar cheese‐derived bioactive peptides (Chingwaru & Vidmar, 2017). During the digestion of casein protein, caseinophosphopeptidases (CPPs) are released owing to strong anti‐tumor capacity against malignant intestinal cells (HT‐29) (Khetra et al., 2016). Calcium accumulated cells are present in caseinophosphopeptidases, where they respond to apoptosis (Perego et al., 2012). Apoptosis occurs due to lactoferricin and β‐casein‐derived peptides in the human leukemic cell line (HL‐60) (Sidira et al., 2017). In an in vivo study, lactoferricin shows cytotoxic activity against cancer cell lines, human leukemic, and neuroblastoma. The α‐casein f (90–95), f (90–96), and β‐casomorphin f (1–5) could suppress the multiple growths of cancer cells lines (López‐Expósito et al., 2017).

### 4.3 Antioxidant peptides

Oxidative stress is a disease condition in which the antioxidant capacity of the cell is reduced. Diseases like cardiovascular ailments, inflammatory, metabolic complications, neurological problems, and cancer are associated with oxidative stress (Irfan et al., 2022; Wolf et al., 2018). Food‐derived antioxidant peptides could appropriately manage oxidative stress (Jiang, Ameer, & Eun, 2020). Milk proteins are considered the best source of antioxidants. Probiotic adjuncts promote the production of antioxidant peptides as compared to non‐probiotic cheese varieties (Jiang, Wu, et al., 2020; Mishra et al., 2015).

An atom or an electron having an unpaired electron is known as a free radical. Oxygen‐free radicals and other peroxyradicals are significant groups of free radicals (Wu, Sun, et al., 2014). Oxidative stress is due to free radicals leading to the formation of tumors and DNA damage (Premanath et al., 2011; Wu et al., 2021). Antioxidants like glutathiones and superoxide dismutase prevent cellular damage in the human body that occupies the free radicals in cells (Huma et al., 2018).

Phenolic amino acids (tyrosine or phenylalanine) are present in Cheddar cheese. They possess antioxidant characteristics (Khan et al., 2023; Maeng et al., 2017). The peptide sequence of Leu‐Leu‐Pro‐His‐His also exerts antioxidant activity in probiotic Cheddar cheese (Kitts & Weiler, 2003).The sequence of the peptide plays a crucial role in deriving highly potential antioxidant peptides, which can be effectively used in several food applications (López‐Expósito et al., 2017).

Nazari et al. (2016) conducted trials on *Lactobacillus acidophilus* (probiotic adjunct) to access total antioxidant power (TAP) using the ferric reducing ability of plasma (FRAP). The study was conducted after 24, 48, and 72 h in bacterial samples and extracts. A significant increase in total oxidant power was observed in *Lactobacillus acidophilus*.

### 4.4 Anti‐hypertensive/ACE inhibitory peptides

The antihypertensive activity of fermented milk products has been studied most comprehensively, both in vivo and in vitro. Antihypertensive peptides are released either as by‐products of fermentation or during proteolysis (López‐Expósito et al., 2017). By calculating the % inhibition of angiotensin I‐converting enzyme (ACE), the antihypertensive activity of peptides could be easily determined in vitro (Nakamura et al., 1995).

The angiotensin‐I‐converting enzyme activity is dependent on chloride ions and also has a degree of dependence on metallopeptidase owing to zinc as a cofactor present in somatic and germinal cells. The angiotensin‐I‐converting enzyme (ACE) is helpful in promoting cardiovascular function and controlling blood pressure in the renin‐angiotensin system (Sieber et al., 2010). Deca peptide angiotensin I is present as an inactive form. Breakdown of angiotensin I occurs to obtain angiotensin II with the help of angiotensin‐1‐converting enzyme (ACE) from the C terminus. Angiotensin II, a vasoconstrictor in nature, can release aldosterone from the adrenal cortex (Liu et al., 2018).

Angiotensin‐1‐converting enzyme inhibition mechanism can influence blood pressure, nervous systems, and regulatory systems of immune defense. In snake venom, the ACE inhibitor was discovered first. Bovine caseins undergo enzymatic hydrolysis, and proteins present in plant and food yield into enzyme inhibitor. Different dairy products obtained through fermentation like cheese, yogurt, and fermented milk are an essential source of ACE inhibitory peptides (Aizawa et al., 2016).

Many of the in vivo and in vitro studies have been conducted to access the antihypertensive activity of Cheddar cheese peptides. Hypertensive rats have been used for the in vivo study. The dairy peptide Val‐Pro‐Pro and Ile‐Pro‐Pro blood pressure‐lowering activity in hypertensive rats has been observed after 6–8 h of dose. A positive impact on blood pressure was observed after taking these peptides for 21 days in clinical studies (Jäkälä & Vapaatalo, 2010; Ur Rehman et al., 2019).

A study was conducted by Ur Rehman et al. (2019) to access the antioxidant and antihypertensive activities of *Lactobacillus acidophilus* and *Bifidobacterium bifidum* in Cheddar cheese. The antihypertensive action of Cheddar cheese was accessed after 60,120, and 180 days of ripening. A significant difference was observed between control and probiotic Cheddar cheese in the inhibition of antihypertensive peptide inhibition. *Bifidobacterium bifidum* showed maximum inhibition.

### 4.5 Immunomodulatory peptides

Immunomodulatory peptides can regulate or suppress the human immune system and could be derived from both casein and whey protein. Some of the examples of immunomodulatory peptides are β‐casein fragments, isracidins, and glycomacropeptides (Megalemou et al., 2017). A positive impact on cell‐mediated immunity was reported in mice because of isracidins released by α_s1_‐casein. Isracidins increased the phagocytosis, uplifted the production of IgG, IgM, and cells responsible for producing antibodies, and increased the lymphocyte production, ultimately improved immunity (López‐Expósito et al., 2017; Quinto et al., 2014).

In the human macrophage cell line (U‐937), glycomacropeptides derived from milk casein improved cell proliferation and phagocytic activities. Peptides of probiotic adjuncts (*Lactobacillus*) boost the production of interleukin‐4 and interferon‐gamma in blood peripheral mononuclear cells (Giromini et al., 2019). LeBlanc et al. (2002) researched the immunomodulatory effect of peptides in milk fermented by probiotic adjunct (*Lactobacillus helveticus*). Increased IgA (+) B cells in mice gut were noted by the administration of fermented probiotic milk peptides. α_s1_‐casein fragments f (1–3), f (55–79), f (101–013), and f (104–105) modulate the immune system. Bioactivity of peptides has been observed because of their necrosis characteristics in healthy mouse T and B cell and human leukemic cell lines. Necrotic cell death may develop a neonatal immune pattern by peptide fragments isolated by trypsin. The peptides can play a significant role in the inhibition of pathogenic infections (Otani & Suzuki, 2003). Pessi et al. (2001) researched suppression of T cell activation by peptides released in bovine Cheddar cheese by probiotic adjunct *Lactobacillus rhamnosus* GG. T cell suppression and reduced proliferation of lymphocytes were observed in vitro by k‐caseinoglycopeptides f (106–109). Reduced lymphocyte proliferation, higher interleukin ten syntheses, and downregulated production of interferon‐gamma and interleukin four were observed by immunomodulatory peptides released by *Lactobacillus paracasei* peptidase hydrolysate (Prioult et al., 2004). Kitazawa et al. (2007) isolated three peptides from β‐casein through activase E and studied macrophage tendency. Increased macrophage tendency was recorded.

### 4.6 Anti‐lipidemic peptides

Hypercholesterolemia and hyperglyceridemia are leading risk factors for cardiovascular issues. Dietary protein can adequately regulate the blood lipid profile. Multiple studies reveal that bioactive dairy peptides are capable of lowering serum cholesterol burden positively. Dairy peptide lactostatin (Ile‐Ile‐Ala‐Glu‐Lys) proved better blood cholesterol regulating activity as compared to β‐sitosterol (a drug used to lower blood cholesterol) in vivo studies (Nagaoka et al., 2001). The cholesterol‐lowering action was further elaborated in vitro studies in human liver cells (Morikawa et al., 2007).

The study was conducted to access the target gene and signal transduction pathway followed by lactostatin in human liver cells. It was observed that lactostatin regulated the phosphorylation of extracellular signal‐regulated kinase (ESK) and intracellular calcium ion concentrations following the MAPK signaling pathway during the lactostatin cholesterol degradation mechanism (Morikawa et al., 2007).

### 4.7 Opioid peptides

Opioid peptides are delivered by casein (β‐casein and β‐lactoglobulin) and whey proteins. Naturally, these peptides are antagonists. These peptides do not need to produce a cellular response to bound receptor sites (Teschemacher, 2003). Morphine‐like effects have been observed from opioid peptides including casomorphins, α‐lactoferrin, and β‐lactoferrin possessing agonistic activity (Ong et al., 2006).

Three types of opioid receptors have been identified. These peptides are capable of binding δ, μ, and κ sites on receptors (Teschemacher, 2003). Opioid peptides exhibiting antagonistic characteristics (lactoferrin and casoxins) are responsible for reducing enkephalins (Rutherfurd‐Markwick & Moughan, 2005). The μ‐type receptor is trapped by casoxin, β‐lactophins, and α‐lactoferrin, while δ‐receptors are trapped by exophorins (Kitts & Weiler, 2003). Structurally, opioid peptides possess N‐terminal tyrosine residues (Ortiz‐Chao et al., 2009). Certain other bioactivities associated with casomorphin are social behavior modulation in infants and animal models, anti‐diarrheal characteristics, and modifying gastrointestinal functionality by preventing gastric drainage (Jia et al., 2021).

Molecular cloning at all receptor points was identified (Pan & Guo, 2010). These receptors are present in the immune, nervous, gastrointestinal, and endocrine systems. Suppression of intestinal motility and emotional behavior is associated with the μ‐receptor. The κ‐receptor is responsible for food intake regulation, and sedation and dynamic behavior are associated with the δ‐receptor (López‐Expósito et al., 2017). In vivo and in vitro studies on therapeutic potential of Cheddar cheese‐derived bioactive peptides are reported in Table 3.

**TABLE 3:** Reported separation and identification techniques of cheese derived bioactive peptides.

Bioactive peptides protein	Bioactive peptides sequence	Separation technique	Cheese varieties	Ripening stage	Subjects of administration	Dosage of intervention	Functional role	Therapeutic potential	References
Peptide A (α_s1_‐CN, B‐8P; f 1–9), Peptide B peptide B (αs_1_‐CN, B‐8P; f 1–13)	Peptide (A) NH2‐Arg‐Pro‐Lys‐His‐Pro‐Ile‐Lys‐His‐Gln‐COOH, Peptide (B) Arg‐Pro‐Lys‐His‐Pro‐Ile‐Lys‐His‐Gln‐Gly‐Leu‐Pro‐Gln, Peptide	RF‐HPLC and gel filtration modes	Gouda cheese	8 months aged	Rats	6.1–7.5 mg/kg of body weight	Anti‐hypertension	Effective in reducing the ACE activity (75.7%)	Saito et al. (2000)
Nisin, Plantaricin	GN=CSN2 PE = 1 SV = 2	Bacterial Hydrolysis by *Lactococcus lactis, Lactobacillus plantarum*	Portuguese cheese	5 days of Fermentation	LAB Species	30 g/L of lactose whey	Anti‐bacterial	90% bacterial growth reduction *L. monocytogenes* and *E. coli* O157	Santos et al. (2021)
β‐Lactoglobulin, kappa casein, lactoferrin	α_s1_‐CN, α_s2_‐CN	RP‐HPLC	Crema de Chiapas (CrC), Fresco (FC)	15 days storage study	Radical scavenging capacity (In Vitro)	1 mg/mL WSE	Anti‐oxidant	High content of amino acid, specifically proline, valine, leucine, phenylalanine exhibit antioxidant properties	Herna (2021)
α_s1_‐CN, β‐CN, β‐CN	EIVPN, KAVPYPQ, PVQPF	RP‐HPLC	Cow milk cheese	NR	In Vitro	7.02–7.63 mg/L	Anti‐oxidant activity	DPPH radical scavenging inhibition, metal chelating activity	Timón et al. (2018)
β‐casein hexapeptides	EAMAPK and AVPYPQ	UHPLC–MS/MS	Stracchino Cheese	NR	Caco‐2 (HTB‐37) monolayer cells	5–150 μg/L	Gastrointestinal protection and bioavailability	Inhibition of ROS, Increased SOD induction	Carmina et al. (2016)
β‐casein and αs2‐ casein	NR	LC Separation and MS analysis	Mimolette and Stilton Blue cheese	21 days	Microplate C18 SPE	20–120 mg/L	Therapeutic potential	Enhancing gut health, reduction in blood pressure, and decrease in modulating inflammation	Robinson et al. (2020)
β‐casein	PR. YPFPGPI peptide	(UHPLC/HR‐MS)	Parmigiano‐Reggiano cheese	18 months	Salivary, gastric and intestinal fluids (SSF, SGF and SIF)	32 μg/L	ACE‐inhibitory activity	Enhancement in bioactivities and different effect of ripening on organoleptic properties of cheese samples	Martini et al. (2020)
Alpha‐S2‐ casein	QYPYQGPIVL	LC‐Q‐TOF‐MS	Goat milk prepared Cheddar cheese	—	HCT‐116 human colorectal carcinoma cell line	10 μg/mL	Anti‐carcinogenic effect	80.92% apoptotic cell death that leads to the promising anticarcinogenic effect	Cakir and Tunali‐Akbay (2021)

Abbreviations: ACE, Angiotensin Converting Enzymes; LCMS, Liquid Chromatography‐Mass Spectrometry; LC‐Q‐TOF‐MS, Liquid Chromatography Quadrupole Time of Flight Mass Spectrometry; NR, Not Reported; ROS, Reactive Oxygen Species; RP‐HPLC, Reversed phase‐High Performance Liquid Chromatography; SOD, Superoxide Dismutase; UHPLC/HR‐MS, Ultra High Performance Liquid Chromatography/High Resolution Mass Spectrometry; UHPLC–MS/MS, Ultra High Performance Liquid Chromatography–Tandem Mass Spectrometry; WSE, Water Soluble Extracts.

## 5 ACTION MECHANISM OF BIOACTIVE PEPTIDES

Lactoferrin, an iron‐binding protein, is present in mammals' secretions. The bacteriostatic nature of lactoferrin depends upon its iron seizing ability. Lactoferricin is associated with lactoferrin. Lactoferrin can suppress the development of parasites, fungi, and Gram‐positive as well as Gram‐negative bacteria (López‐Expósito et al., 2017). The antibacterial activity of lactoferrampin (derived from lactoferrin) against *E. coli, Bacillus subtilis, and P. aeruginosa* in Cheddar cheese was observed by Miladi et al. (2016).

In animal models, orally provided β‐Lg proved an effective peptide having the capacity to inhibit cancer development. β‐Lg owing to good sensory and functional attributes, could be isolated and introduced in functional food and beverages to combat cancer (Chatterton et al., 2006). Bovine serum albumin (BSA), by regulating cell proliferation, positively impacts human breast cancer cell lines in a dose‐dependent way was noticed (Sah et al., 2015).

Antioxidant peptides suppress the aggregation of free radicals, hydrogen peroxide, and other peroxides (Ameer et al., 2017; Fan et al., 2019). Casein is the protein from which antioxidant peptides are predominantly derived. These peptides suppress free radical aggregation by chain breaking, electron acceptor, electron donor, metal deactivator, and UV absorber (Assiri et al., 2016).

Six probiotic Cheddar cheese varieties were prepared to access the antihypertensive activities after 36 weeks of ripening. The probiotic potential was observed to improve the antihypertensive activity during ripening. ACE inhibitory activity increased significantly in all Cheddar cheese samples during the first 12 weeks of ripening. The maximum antihypertensive activity was observed in probiotic adjunct having *Lactobacillus casei* 279 (Rai et al., 2017).

**FIGURE 3:**
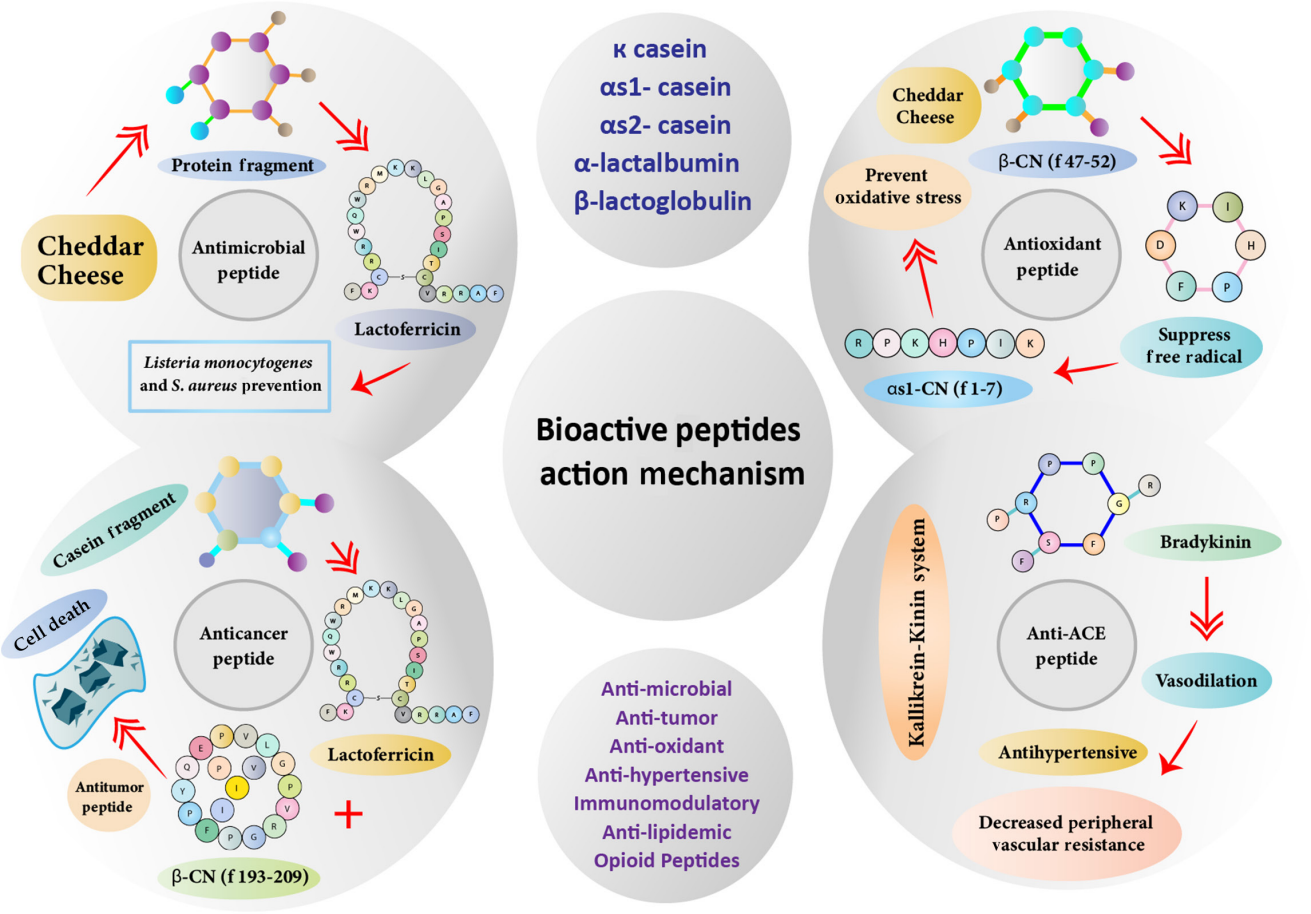
Action mechanism of bioactive peptides.

Aldosterone enhances the graph of blood pressure (Silva et al., 2019). Degradation of bradykinin, substance P, enkephalin, and neurotensin is catalyzed by the angiotensin‐1‐converting enzyme (ACE) in the Kallikrein‐Kinin system. It promotes the functions of the cardiovascular system. Therefore, ACE inhibition is used as a therapeutic approach toward hypertension treatment (FitzGerald et al., 2004). Bioactive peptides provide opioid, anti‐tumor, anti‐lipidemic properties, and immunomodulatory activities. Action mechanism of bioactive peptides is represented in Figure 3.

## 6 CONCLUSIONS

Consumers are more fascinated with naturally produced foods with health‐endorsing and disease‐averting properties since the human race is dealing with numerous health issues. Bioactive substances are the food components that play a positive role in regulating the physiological activities in humans besides the basic nutritional aid. Proteolytic probiotics are commonly used to produce peptides from milk that exhibits anti‐tumor, immune‐stimulatory, opioid, antihypertensive, and ACE inhibitory bioactivity. Probiotic Cheddar cheese is the best example of functional food being rich in bioactive peptides. Keeping the above facts in mind, the review discussed the production of probiotic Cheddar cheese and conclude the impact of probiotics on improving the production and bioactivity of peptides during cheese ripening. This review also highlights the therapeutic potential of bioactive peptides derived from Cheddar cheese.

## 7 FUTURE DIRECTIVE

Milk proteins contain several bioactive peptides that exhibit various curative perspectives. Bioactive peptide content can be enhanced by using suitable methods and techniques. Cheddar cheese contains several bioactive peptides that are considered to have immense therapeutic prospects. Bioactive peptides are inactive within the protein molecule and become active when released by enzymatic hydrolysis of protein, milk fermentation through proteolytic starters, or enzymatic proteolysis. Numerous innovative technologies such as ultrasound, pulsed electric field, and nuclear magnetic resonance can be employed in the production of bioactive peptides. Usage of novel technologies will prevent the limitation that mostly occurs during production with conventional methods. Commercial production of the bioactive peptides should be encouraged by promoting membrane separation and ion exchange chromatographic techniques at an industrial level. Nanotechnology should be applied as a new strategy for the encapsulation of bioactive peptide to maintain stability.

## AUTHOR CONTRIBUTIONS

**Bakhtawar Shafique:** Conceptualization (equal); data curation (equal); formal analysis (equal); investigation (equal); methodology (equal); visualization (equal). **Mian Anjum Murtaza:** Investigation (equal); methodology (equal); visualization (equal). **Iram Hafiz:** Supervision (equal); validation (equal). **Kashif Ameer:** Resources (equal); validation (equal); visualization (equal); writing – review and editing (equal). **Shahnai Basharat:** Validation (equal); visualization (equal); writing – review and editing (equal). **Isam A. Mohamed Ahmed:** Resources (equal); validation (equal); writing – review and editing (equal).

## CONFLICT OF INTEREST STATEMENT

The authors declare no conflict of interest.

## Data Availability

The data supporting the conclusions of this article are included in the manuscript.
